# Molecular Characterization of* Cryptosporidium* spp. in Wild Rodents of Southwestern Iran Using 18s rRNA Gene Nested-PCR-RFLP and Sequencing Techniques

**DOI:** 10.1155/2016/6834206

**Published:** 2016-11-10

**Authors:** Jasem Saki, Masoud Foroutan-Rad, Reza Asadpouri

**Affiliations:** ^1^Health Research Institute, Infectious and Tropical Diseases Research Center, Ahvaz Jundishapur University of Medical Science, Ahvaz, Iran; ^2^Department of Medical Parasitology, Faculty of Medicine, Ahvaz Jundishapur University of Medical Sciences, Ahvaz, Iran; ^3^Department of Parasitology, Faculty of Medical Sciences, Tarbiat Modares University, Tehran, Iran

## Abstract

*Background*. Rodents could act as reservoir for* Cryptosporidium *spp. specially* C. parvum*, a zoonotic agent responsible for human infections. Since there is no information about* Cryptosporidium* infection in rodents of Ahvaz city, southwest of Iran, hence, this survey was performed to determine the prevalence and molecular characterization of* Cryptosporidium *spp. in this region.* Materials and Methods*. One hundred rodents were trapped from different regions of Ahvaz city. Intestine contents and fecal specimens of rodents were studied using both microscopy examination to identify oocyst and nested-polymerase chain reaction (PCR) technique for 18s rRNA gene detection. Eventually restriction fragment length polymorphism (RFLP) method using* SspI* and* VspI *restriction enzymes was carried out to genotype the species and then obtained results were sequenced.* Results*. Three out of 100 samples were diagnosed as positive and overall prevalence of* Cryptosporidium *spp. was 3% using both modified Ziehl-Neelsen staining under light microscope and nested-PCR (830 bp) methods. Afterwards, PCR-RFLP was performed on positive samples and* C. parvum *pattern was identified. Finally PCR-RFLP findings were sequenced and presence of* C. parvum *was confirmed again.* Conclusions*. Our study showed rodents could be potential reservoir for* C. parvum*. So an integrated program for control and combat with them should be adopted and continued.

## 1. Introduction

*Cryptosporidium *spp. are ubiquitous intracellular protozoan parasites that affect wide range of vertebrates like human and livestock [1]. Oocysts are infective stage of life cycle released via feces from infected hosts to environment in large amount. They are resistant to various environmental alterations and remain infective for long period in appropriate condition like surface of water and moist soils; thus, they are liable for transmission of disease to animals and humans [2, 3].* Cryptosporidium *as an opportunistic agent infects the alimentary tract of hosts and have wide spectrum of clinical symptoms in human ranging from self-limiting and asymptomatic in immunocompetent individuals to severe and life-threatening in immunocompromised persons [2, 4, 5]. For first time, human cryptosporidiosis was diagnosed in individuals who had severe watery diarrhea during 1970s [6].

During past decades, numerous evidences have confirmed that small mammals such as rodents are considered as carrier or reservoir for several infectious agents. They can transmit the pathogens directly or indirectly. Also role of rodents in transferring the helminth and protozoan infections are clear [7–10]. Surveys in different zones of Iran indicate the existence of various rodent species such as the house mice (*Mus musculus*), the black rat (*Rattus rattus*), the brown rat (*Rattus norvegicus*), and the Himalayan rat (*Rattus pectoris*) in country [8, 10, 11]. Based on our previous survey in Ahvaz district, southwest of Iran, rodents could be considered as potential source of* Toxoplasma gondii *infection for definitive hosts [9].

Recently, several molecular studies have been conducted to determine the* Cryptosporidium *genotypes. Small subunit-rRNA (SSU 18s rRNA) gene is being utilized for identification of* Cryptosporidium *spp. worldwide extensively. According to SSU-rRNA gene sequencing, at least 20* Cryptosporidium *species have been identified and more than sixty* Cryptosporidium *genotypes have undeterminate status till now. Approximately eight* Cryptosporidium* species/genotypes including* C. parvum*,* C. hominis*,* C. felis*,* C. meleagridis*,* C. ubiquitum*,* C. viatorum*,* C. canis*, and* C. cuniculus* are the main species responsible for human infections, although* C. hominis *and* C. parvum *account for over 90% of human cryptosporidiosis worldwide [1, 2].

Numerous epidemiological surveys have been performed throughout the globe and prevalence of* Cryptosporidium* infection in rodents was highly varied from 63% in UK [12], 32.8% in United States of America (USA) [13], 24.3% in Italy [14], 7.6% in Maryland [15], 11.5% in China [16], 25.8% in Philippines [17], 8.2% in northern Australia [18], 0% in northeast of Iran (Mashhad city) [19], and 27.3% in north of Iran (Tehran city) [20]. Due to lack of reports about* Cryptosporidium* infection in rodents of southwest of Iran till now, current study was aimed to determine the prevalence and molecular characterization of* Cryptosporidium *spp. in this region.

## 2. Materials and Methods

### 2.1. Study Area

Ahvaz city, capital of Khuzestan province which is located in the southwest of Iran (31°50′ N and 49° 11′ E), is ranked as the 7th largest city throughout the country and based on the latest census, its population was calculated at 1,395,184 in 352,128 families. Weather temperature is highly variable throughout the year so that in summer temperature exceeds 50°C whereas in winter it falls to 5°C. Also, annual average rainfall is approximately 230 mm. There is high density of rodents species and rat-man-domestic animals adjacency is remarkable in Ahvaz city [9, 11]. Rodents are additional reservoir for* Cryptosporidium *spp., mostly preyed on by cats and dogs, and, hence, could spread parasitic infections via other animals [8].

### 2.2. Rodents Collection

Ahvaz city initially divided into five geographical locations (north, west, south, east, and center). In each location Sherman live traps were placed outdoor at the entrance of rodent colonies and baited with favorite piece of foods (including cucumber pieces, tomato, and roasted almonds). The Sherman live traps were installed at sunset and gathered before sunrise. Overall, 100 rodents were collected from three different species (6* M. musculus*, 73* R. norvegicus*, and 21* R*.* rattus*). Eventually trapped rodents were gathered and transferred to Department of Medical Parasitology of Ahvaz JundishapurUniversity of Medical Sciences. The trapped rodents were anaesthetized by putting the live traps in a thick transparent polythene bag and then a cotton swab was soaked in ether and placed near their nose. Afterwards, the anaesthetized rodents were dissected and fecal samples gathered from rectum or large intestinal section. Skull and tooth structures were used for species identification. The Iranian rodent key of Etemad was performed to identify the rodents [21].

### 2.3. Detection of* Cryptosporidium *spp. Oocysts

Samples were collected from intestine contents and fecal specimens of rodents. After sugar flotation (SG 1.266; 128 g sucrose and 100 distilled water) [22] and modified Ziehl-Neelsen staining, the samples were examined to find the* Cryptosporidium *spp. oocysts using optical microscope under ×1000 magnification. Diagnosis of oocysts was based on morphological features like red spherical shapes. Finally the samples were maintained at 2.5% potassium dichromate (K_2_Cr_2_O_7_) [23] and kept in refrigerator (1-2 weeks) until DNA was extracted.

### 2.4. DNA Extraction and Nested-PCR

DNA extraction procedure was performed using QIAamp® DNA stool mini kit (QIAamp DNA Stool Mini Kit, USA) based on the manufacturer's guideline. The extracted DNA was kept in −20°C for next tests. For nested-PCR method, we used two specific primers to detect 18s rRNA gene whose length of produced fragments was 1325 bp and 830 bp. This dual stages technique was run using different primers that primary and secondary stages primers were as following: [4, 20, 23, 24].

*First Stage Primers*

Forward (F1): 5′-TTCTAGAGCTAATACATGCG-3′
Reverse (R1): 5′-CCCATTTCCTTCGAAACAGGA-3′

*Second Stage Primers*

Forward (F2): 5′-GGAAGGGTTGTATTTATTAGATAAAG-3′
Reverse (R2): 5′-CTCATAAGGTGCTGAAGGAGTA-3′

Finally PCR products after loading on 1.5% agarose gel and electrophoresis for 1.5 hours were stained with ethidium bromide and then visualized under UV light using Gel Doc device (Uvidoc, Gel Documentation System, Cambridge, UK) [25].

### 2.5. Genotyping* Cryptosporidium *spp. Using 18s rRNA PCR-RFLP and Sequencing

RFLP assay was done to determine the* Cryptosporidium *spp., using digestion of secondary PCR products. For this purpose,* SspI *and* VspI *endonuclease enzymes were employed for species recognition and genotyping, respectively, based on manufacturer's protocol as earlier described [20]. Then the mixture was incubated for approximately 8 hours at 37°C. Eventually visualization of the digested products was carried out under UV transilluminator after 1.5% agarose gel electrophoresis and ethidium bromide staining [20, 26]. The nested-PCR positive samples were purified using Bioneer kit corporation (Korea) and were sequenced by the same corporation. Sequence alignments were constructed by CLUSTAL W software (http://www.ddbj.nig.ac.jp/search/clustalwe.html). Nucleotide sequence data reported in current article are available in the GenBank at accession numbers* AB986579*,* AB986580*, and* AB986581*.

## 3. Results

**Figure 1:**
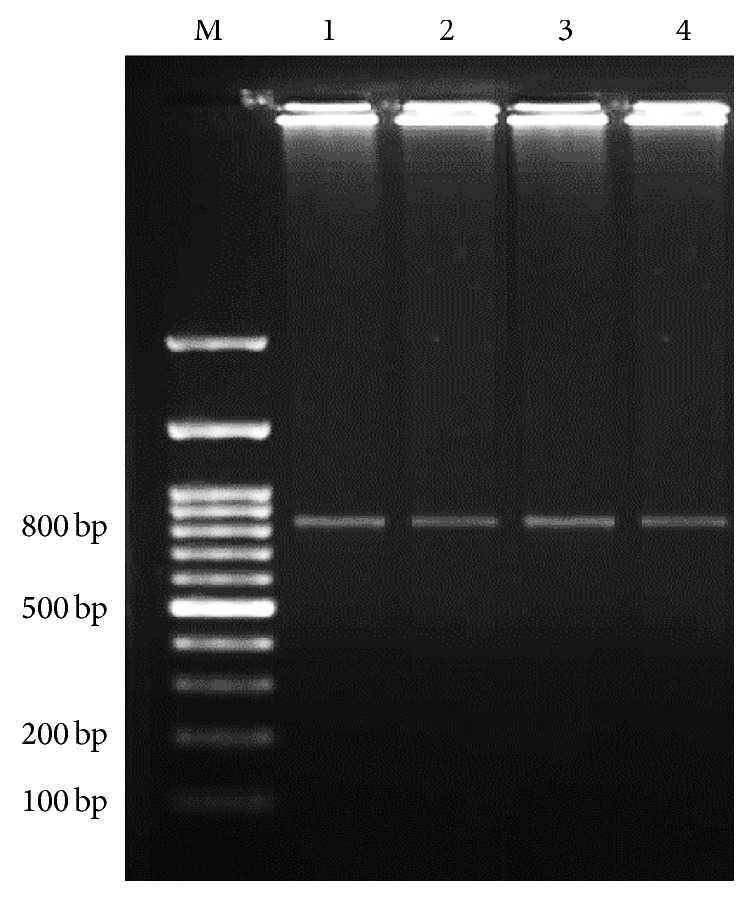
Secondary stage of nested-PCR findings on agarose gel. Lane M, DNA size marker. Lane 1, positive control for* Cryptosporidium*. Lanes 2–4, positive* Cryptosporidium *samples (830 bp).

**Figure 2:**
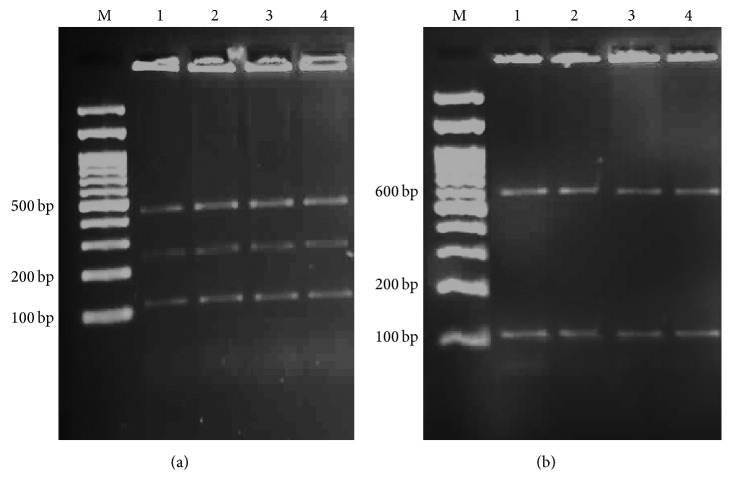
(a) PCR-RFLP products with* SspI *restriction enzyme. Three cuttings in locations 108, 258, and 421 bp are visible on gel electrophoresis. (b) PCR-RFLP products with* VspI *restriction enzyme. Three cuttings in locations 104, 106, and 600 bp are visible on agarose gel. Lane M, DNA size marker. Lane 1, positive control for* Cryptosporidium*. Lanes 2–4, positive* Cryptosporidium *samples.

**Figure 3:**
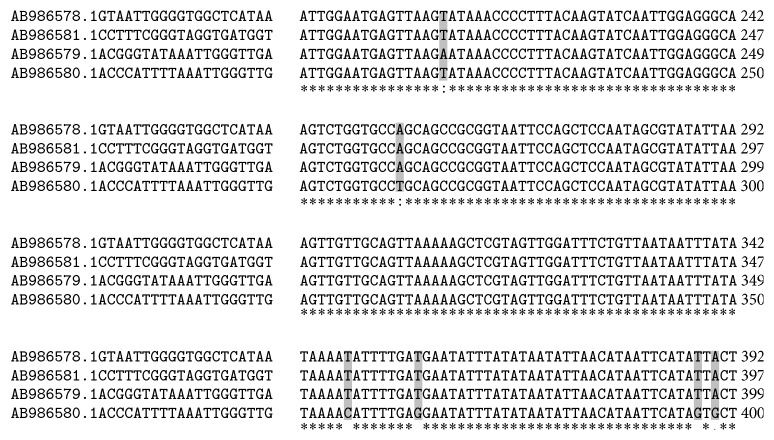
Multiple alignments of 18s rRNA genes from three isolates which submitted to GenBank at accession numbers* AB986579*,* AB986580*, and* AB986581*. Asterisks (*∗*) show identical nucleotides.

Three out of 100 samples were detected as positive for* Cryptosporidium *spp. using modified Ziehl-Neelsen staining under light microscope. In addition, nested-PCR was done and showed 830 bp band which confirmed only 3 samples as* Cryptosporidium *spp. (Figure 1). So, overall prevalence of* Cryptosporidium* infection in rodents of Ahvaz city was calculated at 3% using both methods. All positive samples belonged to* R. norvegicus* (3/73) and from* M. musculus *(0/6) and* R. rattus *(0/21) no positive cases were observed. In order to determine the genotype, PCR-RFLP technique using* SspI *and* VspI *restriction enzymes was utilized. With* SspI *restriction enzyme, three cuttings were seen in locations of 108, 258, and 421 bp visible on agarose gel after electrophoresis. Also after using from* VspI *enzyme, three cuttings happened in locations 104, 106, and 600 bp (Figure 2) and indicate* C. parvum *pattern. The amplified 18s rRNA genes from PCR-RFLP products of three* C. parvum *were sequenced. After submitting the results to the DDBJ/GenBank at accession numbers* AB986579*,* AB986580*, and* AB986581*, the nucleotide sequences were aligned with nucleotide sequences of* C. parvum *certified in GenBank with accession number AB986578 (Figure 3). Based on findings,* C. parvum *was recognized as infectious agent of Ahvaz rodents.

## 4. Discussion

*Cryptosporidium *spp. belong to Apicomplexa phylum with cosmopolitan distribution [1]. Rodents with maintaining the pathogens transmission cycle in surrounding regions play a key role in morbidity and mortality of human and livestock especially in areas with dense population [7].

The routine method for diagnosis of* Cryptosporidium *spp. is based on direct observation of oocysts in stool specimens using optical microscope. Since this method has low sensitivity and needs expertise, also it is unable to distinguish between different species of parasites; thus, molecular techniques like PCR have been used and developed recently. PCR-based techniques with high sensitivity, specificity, and rapidly features are capable of differentiating among species and genotypes in different specimens, that is, water, stool, and animal or human tissues, although they are expensive. Previously, genotyping of* Cryptosporidium *spp. has been done successfully by nested PCR-RFLP method according to SSU-rRNA (18S rRNA) gene [4, 18, 20, 24, 26, 27].

According to previous epidemiological reports, prevalence and rate of infection of* Cryptosporidium *spp. in rodents ranged from 0% to 63% and could be highly variable worldwide [12, 19]. Present investigation is the first report which focused on the prevalence and molecular detection of* Cryptosporidium *spp. in wild rodents of Ahvaz city, southwest of Iran. We found 3% (3/100) prevalence by both direct microscopic observations with Ziehl-Neelsen staining and nested-PCR (830 bp), while, in Bahrami et al. [20] survey in Tehran city (capital of Iran) using these methods, the prevalence was reported at 13% and 27.3%, respectively. Also all positive samples identified* C. parvum *by PCR-RFLP using* SspI *and* VspI *restriction enzymes and were confirmed by sequencing that is in agreement with our study. Differences between Tehran and Ahvaz findings could be justified with sample size, location of sampling, cities population, hygienic conditions, sewage systems, and so forth. It is worth mentioning that Tehran as capital of Iran is the most crowded city with highest density population over the country which is multifold than Ahvaz. In Mashhad city (northeast of Iran), prevalence of* Cryptosporidium *spp. with 0% [19] was lower than our study (3%), while higher prevalence was reported from USA 32.8% [13], China 11.5% [16], Australia 8.2% [18], Philippines 25.8% [17], and Tehran city (north of Iran) 27.3% [20].

Based on current study, 18s rRNA gene of* C. parvum * was detected only in* R. norvegicus *(3/73) and from* R. rattus *and* M. musculus *no positive samples were isolated. It should be mentioned that* R. norvegicus *is the most abundant rodent in southwest of Iran (73/100) and highest prevalence and rate of infection (4.1%, 3/73) allocate to this species which corresponds to previous investigations [9, 11]. The obtained results of amplifying 18s rRNA gene after sequencing were submitted to the DDBJ/GenBank under accession numbers* AB986579*,* AB986580*, and* AB986581* and then compared with AB986578 as control. Figure 3 illustrated several nucleotide replacements.

Lv and colleagues [16] studied the wild, laboratory, and pet rodents (totally 723 rodents from 18 species) in China and prevalence of* Cryptosporidium *spp. was reported at 11.5% and* C. parvum*,* C. muris*,* C. andersoni, *and* C. wrairi *were identified, as well. Prevalence in wild, laboratory, and pet rodents was 6.8%, 1.9%, and 21.8%, respectively. In another survey by Ng-Hublin et al. [17] on 194 wild rats and mice from five species including the Asian house rat (*R. tanezumi*), the rice-field rat (*R. argentiventer*), the Pacific rat (*R. exulans*),* R. norvegicus*, and* M. musculus *in Philippines, overall prevalence was reported at 25.8%. In addition, based on sequencing and phylogenetic analysis of 18s rRNA gene and actin locus,* C. muris*,* C. parvum*,* C. scrofarum*,* C. suis*-like genotype, and rat genotypes I–IV were recognized. In current research, only* C. parvum *was identified using 18s rRNA gene sequencing. Throughout the world* C. parvum *have been isolated from numerous animals such as calves or cattle [24], some ruminants (goats and sheep) [28], horses [29], pigs [30], alpacas [31], some carnivores (gray wolves and dogs) [32, 33], reptiles [27], and rodents (rat, hamster, mice, nutria, chipmunk, squirrel, and capybara) [13, 16, 17, 20, 34–37]. In addition predominant* Cryptosporidium *species in Iran is* C. parvum* that has been verified frequently, for example, 73.3% in humans and animals [38], 83.3% in Tehran (human samples) [39], 100% in rodents of Tehran [20], 100% in cattle of Ilam [24], and 68.8% in Ahvaz (in immunocompromised patients and children) [4]. In Rafiei et al. investigation in southwest of Iran on immunocompromised patients and children (kidney transplant recipients, persons with hematological malignancies, HIV+ patients, and children less than 5 years old), 390 stool specimens were collected and examined. Prevalence of* Cryptosporidium *spp. was 4.1% (16/390). Moreover, 3 different species were identified using PCR-RFLP based on 18s rRNA gene including 11* C. parvum*, 4* C. hominis*, and 1* C. meleagridis *[4] which was in consistent with our results. According to Rafiei et al. [4] survey and our study,* C. parvum *was identified as the most common species in both rodents and individuals in Ahvaz city. In past studies* C. parvum* transmission from rodents to human was reported repeatedly [17, 40].

## 5. Limitations

The present investigation was based on sampling of limited rodents species in limited areas. In future, for better understanding of exact burden of* Cryptosporidium *spp. and genetic diversity, studies should be designed on wide spectrum of both wild and pet rodents (rats, hamsters, mice, rabbits, etc.) in vast regions like throughout the Khuzestan province.

## 6. Conclusion

Present paper was the first report which focused on the prevalence and molecular characterization of* Cryptosporidium *spp. in wild rodents of Ahvaz city, southwest of Iran; that showed rate of infection in* R. norvegicus *is remarkable. Also, rodents could be potential reservoir for* C. parvum*. The results can help public health care to pursue new strategies (environmental sanitation, increasing the hygienic condition, increasing the hygienic condition, etc.). In future, adopting a suitable strategy for control and combat with rodents in order to decrease human cases is necessary and should be continued.
